# It’s not just the antibiotics, it’s the treatment

**DOI:** 10.1186/cc13904

**Published:** 2014-06-03

**Authors:** Christopher A Guidry, Robert G Sawyer

**Affiliations:** 1 Department of Surgery, The University of Virginia Health System, Charlottesville, VA 22908-0679, USA; 2 Division of Acute Care and Trauma Surgery, The University of Virginia Health System, Charlottesville, VA 22908-0679, USA

## Abstract

The recent study by Bloos and colleagues demonstrates that early initiation of antimicrobial therapy is not associated with improved survival in sepsis. We contend that these findings should not be surprising. This study is yet another part of the growing case against early and aggressive antimicrobial therapy and highlights the important roles resuscitation and source control play in the management of the septic patient. We suggest that, whenever possible, antimicrobial therapy should we withheld until objective evidence of infection has been obtained.

## 

Bloos and colleagues [1] present the results of a large, prospective, multicenter, cohort study of patients in severe sepsis or septic shock. The authors evaluated 1,011 medical and surgical patients. They identified that, in all cases, delay of antimicrobial therapy of more than 1 hour was not independently associated with mortality. In the surgical cohort, a delay in source control greater than 6 hours was significantly associated with mortality. For the combined cohort, inappropriate therapy was also associated with mortality on multivariate analysis. These results are compelling and go against conventional wisdom on this subject.

In studies of this type it is often difficult to discern the true time of infection. This study is no different. The authors have chosen to use the time of first infection-related organ dysfunction. While we appreciate its objective nature, this definition presents a two-fold problem. Firstly, 57.4 % of patients developed sepsis in the ICU. Presumably, most ICU patients were there in the first place because of some underlying organ dysfunction. It may not be immediately apparent that this dysfunction is infection-related, making this a definition that can only be applied retrospectively. For example, how did the authors handle ventilator-associated pneumonia, where patients by definition already had failure of one organ system? Secondly, 13.9% of patients 'developed' sepsis in the emergency department. Therefore, the time until initiation of therapy is more indicative of the time from arrival until recognition of sepsis than the true time from onset of organ dysfunction. These patients were likely suffering organ dysfunction upon arrival to the emergency department. It would be interesting to see the results with these patients excluded.

Should these findings have been surprising? We think not. The author’s cite a meta-analysis by Barochia and colleagues [2] that suggests that timely administration of antimicrobials is associated with improved survival. However, this finding was only significant after removing the effects from Rivers and colleagues’ study on early goal-directed therapy [3]. Another meta-analysis conducted on studies of outpatient respiratory infections found no difference between immediate or delayed antibiotic therapy [4]. Puskarich and colleagues [5] demonstrated that, in the setting of appropriate resuscitation, the timing of antimicrobial therapy appears to have little effect on mortality. Similarly, Hranjec and colleagues [6] found that, assuming resuscitation and source control occur in a timely manner, intentionally withholding antimicrobial therapy until infection can be objectively proven to result in improved patient outcomes compared with early and aggressive initiation. These studies focused on emergency department and ICU management, respectively. Bloos and colleagues’ study confirms the findings of Hranjec and colleagues and Puskarich and colleagues, while spanning the entire spectrum of hospitalization.

This trial also suffers from the most common deficit in most studies of timing of antimicrobial therapy in patients with severe sepsis or septic shock: the lack of a proper control group. The question faced by the clinician is not whether a patient with proven infection should have the rapid initiation of antimicrobials, but whether a patient who might be infected should receive therapy. Missing in this study is the group of patients who received antimicrobials for suspected infection but were not infected, and what the consequences of this unnecessary exposure to antibiotics were. However, the data are reassuring to us who believe that it is prudent to wait until there is objective microbiological data to support the diagnosis of infection before initiating therapy. Even among these patients with significant disease, the exact timing of antimicrobial therapy did not appear to be the main driver of outcomes.

At its core, the narrow-focused debate surrounding the timing of antimicrobial therapy is inherently flawed. The appropriate treatment of the septic patient requires a rapid multimodal response including fluid resuscitation, source control, and pressors (if required). Antimicrobials are only one part of this equation. In cases of life threatening septic shock, broad-spectrum empiric therapy should be initiated as soon as possible. However, the harmful effects of inappropriate antimicrobials should be mitigated, whenever possible, by using culture-guided therapy and delayed initiation. In all cases, resuscitation and source control should be the highest priority. Bloos and colleagues’ study is a reminder that antimicrobials, while important, are not the only tools in our arsenal against infectious disease.

## Conclusion

Overall, Bloos and colleagues have provided another interesting wrinkle in the story of antimicrobials and sepsis. Their study is well executed and practical, and the authors are to be commended for their excellent work. We look forward to the results of their randomized trial.

## Competing interests

The authors declare that they have no competing interests.

## Authors’ contributions

Both authors drafted, made critical revisions, read, and approved this manuscript.
